# Challenges and opportunities for effective adoption of HRH information systems in developing countries: national rollout of HRHIS and TIIS in Tanzania

**DOI:** 10.1186/s12960-015-0043-1

**Published:** 2015-06-16

**Authors:** Hisahiro Ishijima, Martin Mapunda, Mathew Mndeme, Felix Sukums, Violeth Solomon Mlay

**Affiliations:** HRH Development Project, Japan International Cooperation Agency, Dar es Salaam, United Republic of Tanzania; Ministry of Health and Social Welfare, Dar es Salaam, United Republic of Tanzania; Department of Human Resource Development, Ministry of Health and Social Welfare, Dar es Salaam, United Republic of Tanzania; Department of Computer Science and Engineering, University of Dar es Salaam, Dar es Salaam, United Republic of Tanzania; EnterSoft Systems Ltd, Dar es Salaam, United Republic of Tanzania; Mkulanga District Hospital, Mukuranga, United Republic of Tanzania

**Keywords:** Human Resource for Health Information System (HRHIS), Training Institution Information System (TIIS), Health training institutions, National rollout, United Republic of Tanzania

## Abstract

**Background:**

The establishment of a functional information system for human resource for health (HRH) was one of the major challenges for the Tanzanian health sector. In 2008, the Ministry of Health and Social Welfare developed the HRH Strategic Plan, in which establishment of computerized information systems were one of the strategic objectives. In response to this objective, the Ministry developed two information systems, namely the Human Resource for Health Information System (HRHIS) and the Training Institution Information System (TIIS), to capture information from both the public and private sectors.

**Case description:**

The national rollout of HRHIS and TIIS was carried out in four phases during a 6 year period between 2009 and 2014. Together with other activities, the rollout process included conducting system operation training and data utilization training for evidence-based planning, development and management of HRH and social welfare workers and health training institutions.

**Discussion:**

HRHIS was rolled out in all 25 regions of the Tanzanian mainland, including 171 districts, and TIIS was rolled out in all 154 health training institutions and universities. Information is captured from both the private and public health sectors with high-data coverage. The authors identified several key factors for the achievements such as using local experts for developing the systems, involvement of system users, positive attitudes among users, focusing on routine work of the system users and provision of operations and data utilization trainings. However, several challenges were also identified such as getting a consensus on sustainable HR information systems among stakeholders, difficulty in obtaining baseline HRH information, inadequate computer skills and unsatisfactory infrastructure for information and communication technology. We learned that detailed situation analysis and understanding of the reality on the ground helped to reduce the “design–reality gap” and contributed to establishing user-friendly systems and to improve sustainability of the systems.

**Conclusions:**

This paper illustrates the successful development and national rollout of two information systems for HRH in Tanzania. The approaches used and activities conducted here and lessons learned could be useful for countries which are planning to establish HR information systems.

## Introduction

During the first global forum for Human Resource for Health (HRH), held in Kampala, Uganda, in 2008, part of the Kampala Declaration and Agenda for Global Action [1] included the establishment of health workforce information systems for the improvement of research activities. It also aimed to increase the capacity of data management for evidence-based decision-making and to enhance shared learning. However, having functional HRH information systems to obtain reliable information for evidence-based HRH planning, development and management remains a major challenge in low-income countries.

The World Health Organization (WHO) called a technical meeting to strengthen health workforce information systems in 2010. The aim of the meeting was to initiate discussion on how to promote a coordinated, harmonized and standardized approach to strengthening country health workforce information and monitoring systems to support policy, planning and research [2]. Based on the meeting report, it was reported that health workforce information and monitoring systems in low-income countries tend to be unreliable with poor linkages to other data sources. Weak management of administrative HRH data on each of the stages of the working lifespan and weak analysis and utilization of HRH information to HRH policy and strategies were also pointed out [2]. Additionally, low competency of staff handling computerized information systems or insufficient competent workers to operate information systems were pointed at as being one of the major bottlenecks on sustainability of computerized information systems [2-4].

WHO made a series of recommendations to overcome the challenges regarding establishment and utilization of an HRH information system. The capacity building of health personnel in collection, management, analysis, interpretation and use of HRH data; the need to collect HRH data from multiple sources; the advantage of interoperability with other systems; and strengthening the use of HRH data for policy, strategies and guidelines were recommended by WHO. Those recommendations are also seen in different studies and reports [2, 5, 6]. Tanzania was one of the countries that was keen to establish functional, effective computerized HRH information systems for better planning, development and management at all levels because the Ministry of Health and Social Welfare (MOHSW) experienced the failure of health information systems in the past [7].

The first information system for the health sector was the Health Management Information System (HMIS) Version 1, which was introduced from 1994 to 1997. The HMIS captured HRH information in the early stages of the health sector reform in Tanzania. Additionally, brief information on “Personnel Database and Human Resource Development Database (HRD)” is given in the Tanzania Joint Health Technical Review 2002 HMIS Sub-Group Final Report [8].

There was no HRH-specific information system, and therefore, the MOHSW tried to capture HRH information from different information systems operated by other ministries in the country. However, a weakness in information collection and data analysis for HRH demand and forecasting was pointed out in the joint external evaluation report [7]. Therefore, a need for the establishment of a functional and comprehensive HRH information system was advocated as a priority area in a number of policy documents within MOHSW [7].

Based on the critique and needs from various stakeholders in the health sector, MOHSW developed the HRH Strategic Plan (HRHSP) 2008–2013 in 2008, with the composition of seven strategic objectives. Strategic Objective number one (SO1) was to “Strengthen human resource planning and policy development at central level”. One of the key strategies to achieve SO1 was “Establishment of comprehensive HR Information Systems at all levels” [9].

Due to the financial requirements of establishing HRH information systems, MOHSW made an official request to the Japan International Cooperation Agency (JICA) to support the development of system application software and the national rollout of the HRH information systems. The request from the Government of Tanzania was accepted in 2008 [10].

During the process of establishing HRH information systems, challenges mentioned in the WHO report 2010 [2] were also observed in Tanzania. However, MOHSW succeeded in overcoming those challenges and rolled out two HRH information systems nationwide. This case study describes how MOHSW overcame those challenges and accomplished national rollout of the HRH information systems. The definition of “success” of information systems can be argued. Various studies have tried to establish criteria for assessing the success of information systems based on various independent variables. The success of IS has been regarded as dependent, variable and complex to establish since the contributing factors to the success of the system are many, multidisciplinary by nature and interrelated [11].

Heeks’s study [12] defined “success” of an information system as “an initiative in which most stakeholder groups attain their major goals and do not experience significant undesirable outcomes” [13]. DeLone and Mclean [14] argued that the information system will be regarded as successful based on criteria to do with the technical success of the system which is measured by the system quality, semantic success which is measured by information quality and effective success which is measured by use and user satisfaction, and the net benefit of the system to the users and the organization. In this article, the authors regard the “success” of the information system as “the information system is understood, adopted, and utilized by the intended users at each level”. Furthermore a “success” of a software project relatively depends on the perception of the evaluators. While some regard cost and time as vital determinants of software project success, others focus on functionality and quality of the project outcomes [15].

## Case description

**Fig. 1:**
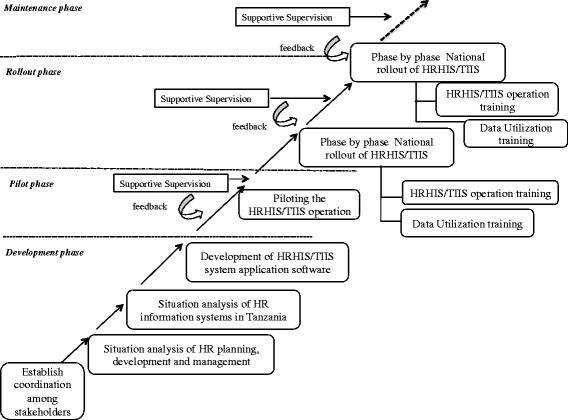
National rollout process for HRHIS and TIIS

The development and rollout of two HRH information systems, namely the Human Resource for Health Information System (HRHIS) and Training Institution Information System (TIIS), were carried out in phases. The four phases are 1) development phase, 2) pilot phase, 3) rollout phase and 4) maintenance phase, as visualized in Fig. 1.

### Development phase

The system development started in 2008. After MOHSW presented HRHSP 2008–2013, many implementers, who were supported by different donors, brought their system application software to MOHSW. The implementers demonstrated their system applications and explained that their information systems were the solution to HRH problems and appealed for their adoption. However, MOHSW was not convinced by the solutions presented by these implementers and decided to use the available local resources in Tanzania to establish local solutions. This was done to avoid design–actuality challenges experienced in adoption of previous information systems which had been designed by external organizations and posed various limitations like lack of technical expertise for maintenance, inflexibility of the software together with high costs for maintenance and updates of the software.

The Department of Human Resource Development (DHRD) of MOHSW organized a series of workshops on coordination and development of their own HRH information systems. Relevant stakeholders were identified and invited to the workshops to discuss the outlines of HRH information systems. Data elements to be collected, information flow, the data collection method, confidentiality, communication with other information systems and other basic issues were discussed and agreed on through the workshops. Then, situation analysis was conducted to clarify the administrative work regarding HRH planning and management at different levels of the government to verify the issues discussed in the workshop. Existing human resource (HR) information systems in the country were also studied to establish interoperability approach. Based on the results of the detailed situation analysis, the system application software for HRHIS and TIIS was designed and developed.

#### Description of the HRHIS and TIIS

**Fig. 2:**
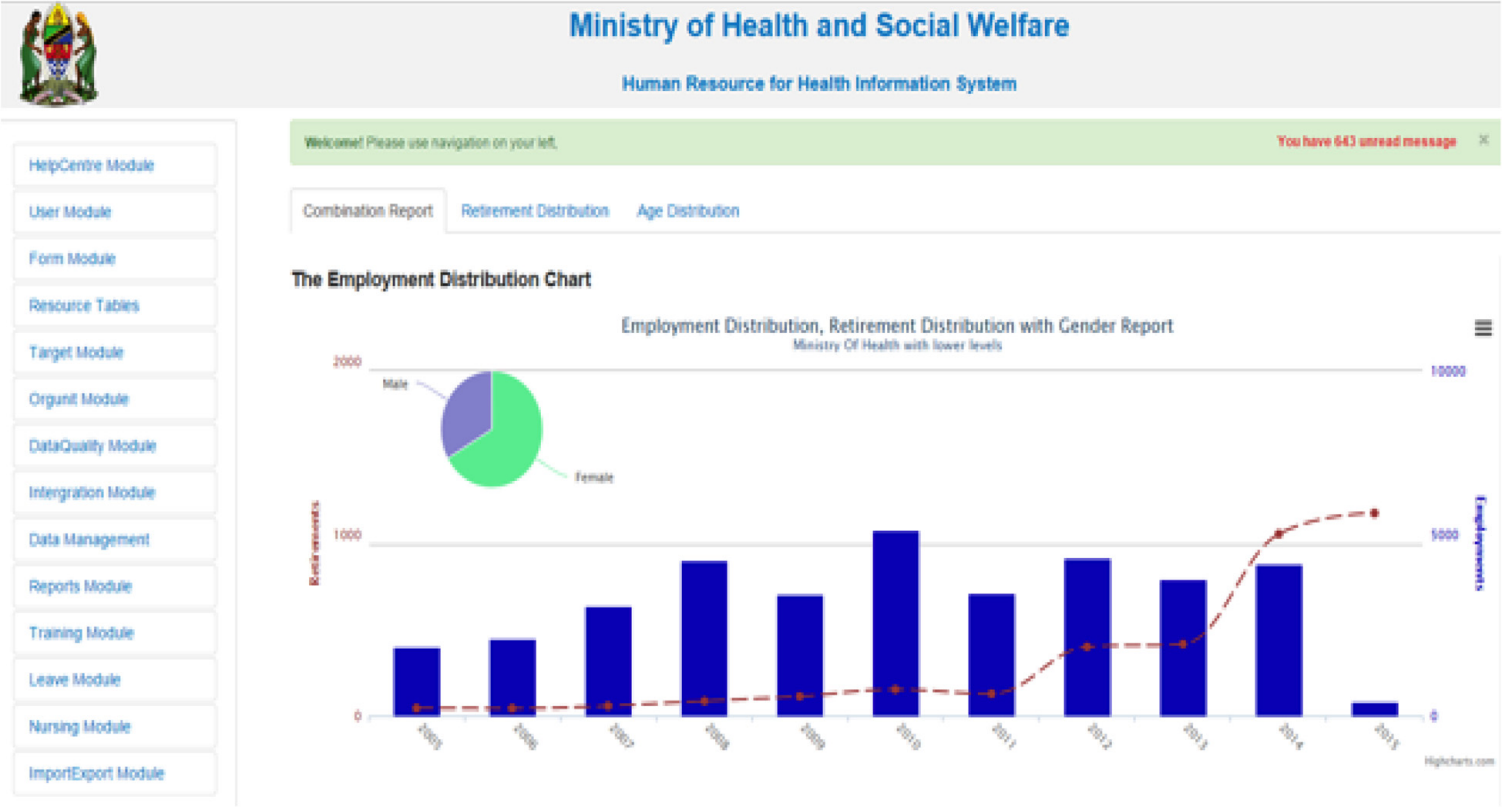
The HRHIS dashboard showing its modules

HRHIS is a web-based software that enables a health system to collect, validate, analyse and present raw and statistical human resource information for reporting, analysis and decision-making. It is a generic software tool that allows customization to fit organization-specific requirements, and it is built with open metadata models and a flexible customizable user interface that allows a user to adjust the system to perform, behave, look and feel based on the organization’s specific requirements without the need for software development expertise. It is based on framework-dependency injection and event dispatchers, which therefore makes it flexible, customizable and extensible in a way that more modules can be plugged in with simplicity. HRHIS uses web application programming interfaces (APIs) to interoperate by third-party applications to share the specified set of data. It collects human resources data from public, private and faith based organizations’ health facilities. Reports generated from the system can be exported in different formats like excel, image and PDF, therefore giving flexibility of using and sharing based on demand and audience. Example screenshots are shown in Fig. 2.

**Fig. 3:**
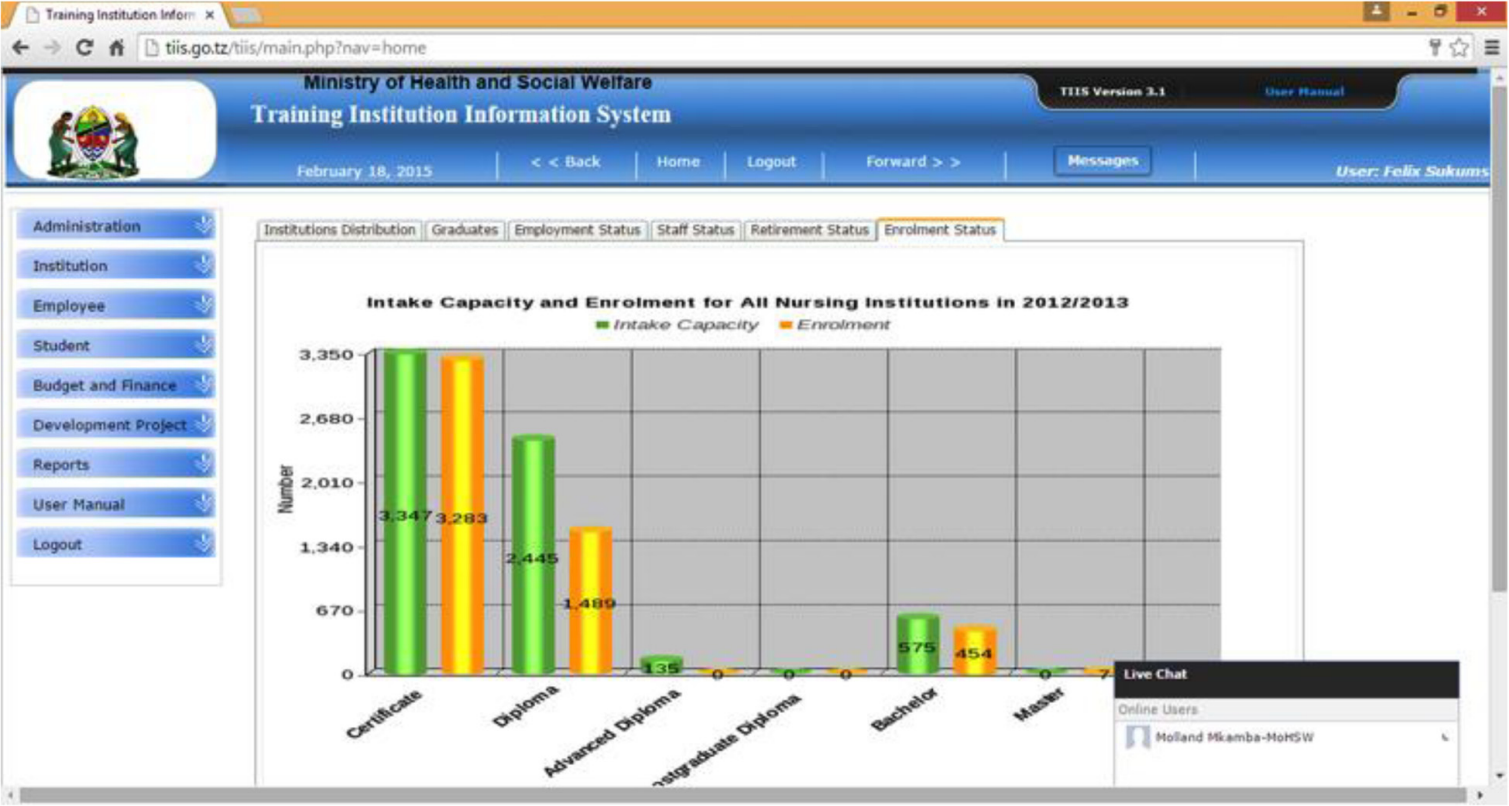
The TIIS dashboard showing its modules

TIIS is a web and enterprise information system for health training institutions in Tanzania Mainland, which facilitates information capturing, analysis and informed decision-making during planning, management and development of human resource for health and institutions’ resources. The system has several modules and functions for managing information about institution, academic programme and courses, student academic records (continuous assessments and examinations), employees, assets, development projects and budget and finance. Other features include administration and user management, public portal, data management and reports, and API for data sharing with other systems. Example screenshots are shown in Fig. 3.

### Pilot phase

In the pilot phase, from the end of 2008 to 2009, HRHIS was piloted in 10 districts within two regions, and TIIS was piloted at 22 training institutions located in three different regions. During the pilot phase, system operation trainings were conducted at the pilot sites. Use of the two systems was monitored through supportive supervision. Functions of the two system applications, data collection tools, collection mechanism and reporting on HRH data were tested.

At the beginning, some facilities, regions, districts or training institutions were not cooperative with the data collection process, and private facilities were not willing to share HRH data. After detailed explanation and advocacy of the systems, pilot sites accepted the systems.

Several issues were identified during the pilot phase, including weak Internet connectivity, poor computer literacy and instability and insufficient power supply. In addition, some basic data elements to facilitate HRHS management activities such as profession, employment history and in-service trainings were not always needed for HRH management at the district level, while other elements such as personal details, employment status, salary scale and duty post and designation were demanded. This situation was also caused by users who were confused between data that needed to be recorded into the system and those which could be calculated automatically by the system such as retirement date and age.

The first version of HRHIS was piloted with 17 fields as a minimum dataset. However, since the system had been developed and deployed using user participation and iterative approach, the minimum dataset has been periodically revised and improved to the current one with 32 fields. These data fields include employees’ personal details, education and professional details, contacts information, employers’ information, employment history and status, in-serving trainings, salary scales and duty post.

Flexibility to modify data collection elements, the appearance of reports and other user needs obtained from the pilot phase were studied and reflected on for improvement of the system application software to increase user friendliness. The findings from the pilot phases were also utilized for modification of the strategy and approach before rollout of HRHIS and TIIS. Upon completion of the pilot phase, rollout of HRHIS and TIIS began (Table 1).

**Table 1:** Data elements collected for HRHIS and TIIS employees module

Sq no.	Data elements	Sq no.	Data elements
1	First name	17	Profession
2	Middle name	18	Present designation
3	Surname	19	Superlative substantive position
4	Date of birth	20	Department
5	Sex	21	Salary scale
6	Marital status	22	Monthly basic salary
7	Nationality	23	Date of first appointment
8	Religion	24	Date of confirmation
9	Basic education level	25	Date of last promotion
10	Profession education level	26	Employer
11	Number of children/dependents	27	Employment status
12	District of domicile	28	Registered disability
13	Check number	29	Contacts of employee
14	Employer’s file number	30	Next of kin
15	Registration number	31	Relationship to next of kin
16	Terms of employment	32	Contacts of next of kin

### Rollout phase

The systems were rolled out from the middle of 2009 to the beginning of 2014 [16]. The rollout phase was divided into five subphases. The Plan-Do-Check-Act (PDCA) cycle [17] was applied to improve the systems, rollout strategies and activities by subphases based on the needs of the users.

During the rollout phase, several interventions were carried out to ensure proper handling of HRH information and data use for planning, development and management of HRH and health training institutions within the country at all levels.

#### Operation training

Target groups of HRHIS operation training were the district/council health management teams (CHMTs), regional health management teams (RHMTs) and large hospitals with many employees. The reason for targeting CHMTs and RHMTs is because CHMT is responsible for recruiting and management of HRH and social welfare staff for district-level health facilities. RHMT is responsible for recruiting and management of HRH and social welfare staff for the regional referral hospital.

Target groups of TIIS operation training were training institutions and universities that produce health professionals. Key personnel from the target groups were called to participate in the operation training of the systems.

**Fig. 4:**
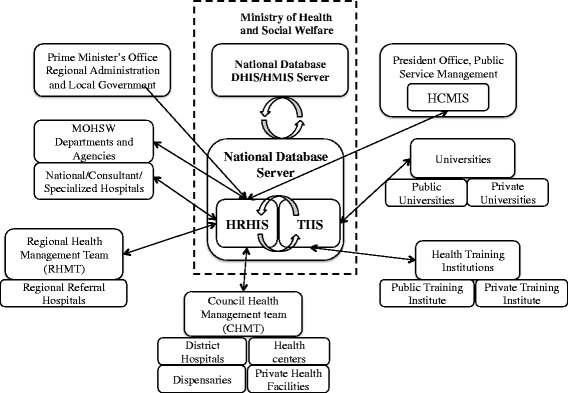
Relations among different organizations and HRHIS/TIIS

A 3 day intensive training workshop was conducted to learn how to operate the systems. Since HRHIS and TIIS are computerized information systems, basic computer skills were taught at the beginning of the training, followed by operational skills and knowledge of the systems. All training sessions were conducted at regional capitals. However, the arrangement of computer laboratories in remote areas was challenging. Therefore, laptop computers, projectors and associated accessories were procured for a mobile computer laboratory, and both systems were installed onto the computers. This mobile training computer laboratory was very useful in training many target users within a short period and in reducing lots of logistic arrangements, as well as costs for hiring computer laboratories (Fig. 4).

#### Supportive supervision of HRHIS/TIIS

Supportive supervision visits of the utilization of HRHIS and TIIS were conducted twice a year. Supportive supervision teams were formulated by MOHSW staff and system developers, and they visited all the sites where HRHIS and TIIS were introduced. RHMTs were also involved in supportive supervision of HRHIS. RHMTs were very helpful and had significant contribution on the implementation of the supportive supervision, such as providing a vehicle for transport to districts for supportive supervision. Zonal training coordinators also contributed a lot to TIIS in terms of communication and follow-up of TIIS usage.

**Fig. 5:**
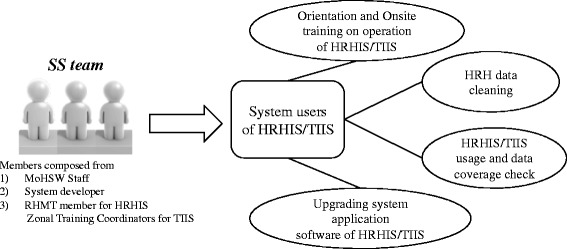
Supportive supervision of HRHIS/TIIS

During the supportive supervision, a standardized monitoring sheet was used to check the progress of data entry and system use. Aspects like the information and communication technology (ICT) environment, activities of trained personnel, data entry and updating and utilization of information generated from the systems were monitored. Additionally, if a weakness in system usage was observed, the supportive supervision team provided onsite training to capacitate the system users. If the system was upgraded, upgrading of the system application was also carried out during the supportive supervision. Recently, both HRHIS and TIIS were modified to make online software upgrade possible. Whenever the system users are connected with a reliable Internet connection, they are able to register or obtain necessary HRH information anytime and anywhere. Moreover, both systems established online technical support, which can upgrade software as well as supporting the users in operation of HRHIS and TIIS though the messaging modules created within the systems (Fig. 5).

#### Data Utilization Training (DUT)

During the situation analysis, it was found that many health managers at the regional and district level and at training institutions did not have enough knowledge of HR planning, development and management. Therefore, MOHSW developed guidelines for HRH data utilization for the two systems.

MOHSW formulated two teams with five experts per team and used the guidelines to train health managers on HRH planning, development and management. Pre- and post-assessments were conducted to measure improvement of knowledge of HR planning, development and management. Additionally, the effect size (*d*) was calculated to measure effectiveness of the training. Effect size (*d*) was calculated from the average of the post-training assessment scores minus the average of the pre-training assessment scores divided by the standard deviation of the two conditions [18,19].

In 90 % of data utilization trainings (DUTs), there is a statistically significant increase in knowledge after the training (*P* < 0.01). Twelve DUTs out of 20 had large-effect sizes (Δ0.80 ~ Δ1.45), and 6 DUTs out of 20 had middle-effect sizes (Δ0.51 ~ Δ0.69). One DUT showed a low-effect size (0.30) (Table 2).

**Table 2:** Result of pre-post-assessment of data utilization training

DUT by regions		Mean	Std. dev.	95 % CI		*T*-test	*P* value	Effect size	Δ score
				Low	High				
Arusha	Pre	36.48	4.00	35.0	37.9	−7.762	<0.001	1.15	|.80| ≦ large
	Post	41.09	3.00	40.0	42.1				
Dar es Salaam	Pre	66.42	13.67	62.0	70.7	−5.291	<0.001	0.80	|.80| ≦ large
	Post	77.40	9.35	74.4	80.3				
Dodoma	Pre	66.10	13.08	59.7	72.4	−3.195	<0.005	0.83	|.80| ≦ large
	Post	76.94	8.87	72.6	81.2				
Iringa	Pre	63.72	10.57	59.6	67.7	−4.837	<0.005	0.86	|.80| ≦ large
	Post	72.82	7.51	69.9	75.6				
Kagera	Pre	71.09	10.19	67.9	74.1	−9.752	<0.001	1.20	|.80| ≦ large
	Post	83.27	6.94	81.1	85.3				
Kilimanjaro	Pre	45.32	29.46	38.3	52.3	−3.829	<0.001	0.56	|.50| < medium < |.80|
	Post	61.80	25.08	55.8	67.7				
Kigoma	Pre	68.80	12.71	38.3	52.3	−3.829	<0.001	0.81	|.80| ≦ large
	Post	79.06	9.60	55.8	67.7				
Lindi	Pre	64.34	9.52	61.2	67.4	−5.801	<0.001	0.89	|.80| ≦ large
	Post	72.76	7.71	70.2	75.3				
Manyara	Pre	74.96	10.71	70.7	79.2	−6.460	<0.001	0.67	|.50| < medium < |.80|
	Post	82.11	6.80	79.4	84.8				
Mara	Pre	66.32	11.98	61.9	70.7	−3.549	<0.001	0.51	|.50| < medium < |.80|
	Post	72.38	10.60	68.4	76.2				
Mbeya	Pre	67.86	9.48	64.3	71.4	−4.648	<0.001	0.80	|.80| ≦ large
	Post	75.40	6.37	73.0	77.7				
Morogoro	Pre	54.15	23.48	46.6	61.6	−10.263	<0.001	0.30	|.20| ≦ small < |.50|
	Post	61.17	23.91	53.5	68.8				
Mtwara	Pre	70.00	10.53	64.3	75.6	−2.953	<0.010	0.69	|.50| < medium < |.80|
	Post	77.25	6.48	73.7	80.7				
Mwanza	Pre	67.76	13.85	64.3	71.1	−6.639	<0.001	0.56	|.50| < medium < |.80|
	Post	75.52	10.68	72.9	78.1				
Rukwa	Pre	65.20	7.57	61.6	68.7	−2.972	<0.010	0.93	|.80| ≦ large
	Post	72.20	9.40	67.8	76.5				
Ruvuma	Pre	68.75	9.26	63.8	73.6	−2.226	0.041	0.57	|.50| < medium < |.80|
	Post	74.00	9.35	69.0	78.9				
Shinyanga	Pre	68.48	11.95	63.9	73	−8.705	<0.001	1.20	|.80| ≦ large
	Post	82.75	7.27	79.9	85.5				
Singida	Pre	70.10	10.53	66.0	74.1	−8.428	<0.001	1.45	|.80| ≦ large
	Post	85.34	8.26	82.1	88.4				
Tabora	Pre	NA	NA	NA	NA	NA	NA	NA	NA
	Post	NA	NA	NA	NA				
Tanga	Pre	60.70	13.97	53.5	67.8	−4.428	<0.001	0.86	|.80| ≦ large
	Post	72.76	6.84	69.2	76.2				

#### Data coverage

HRH information was captured from both the private and public health sectors. Data coverage of HRHIS was 94 % in the public sector and 83 % in the private sector, increasing from 85 % and 56 %, respectively, recorded at the time of midterm review in November 2012.

**Fig. 6:**
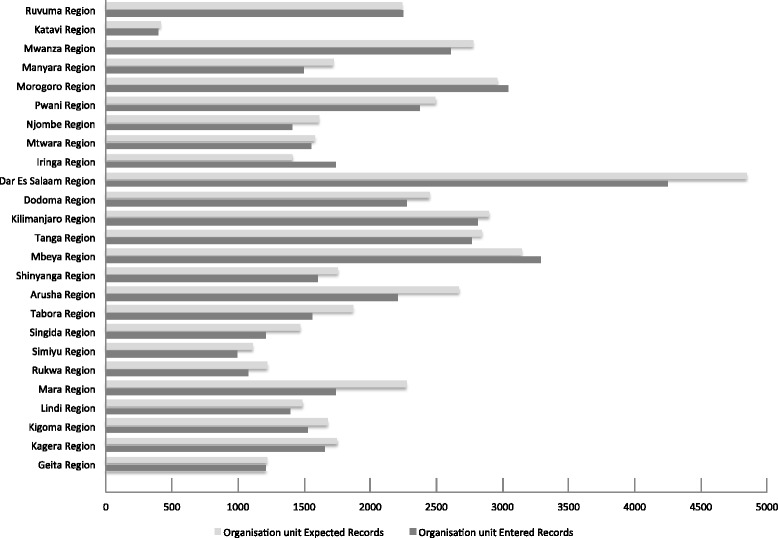
HRHIS data coverage

**Fig. 7:**
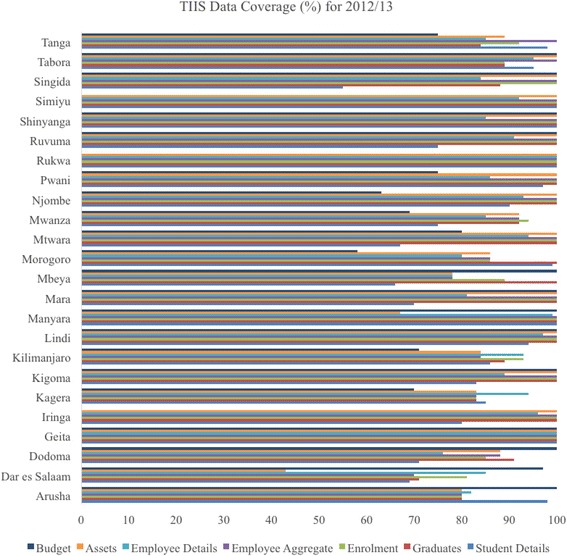
TIIS data coverage

In Fig. 6, the HRHIS data coverage rate in the public sector by region is explained, and Fig. 7 explains the TIIS data coverage rate in the public sector by region. The data coverage rate was calculated using the information obtained both from counting and from existing papers: the number which was entered into HRHIS through physical counting was the numerator, and the denominator was the number counted from existing staff paper files or records. Some regions show more than 100 % data coverage. This was caused by the gap between actual information entered into HRHIS and paper files of HRH and social welfare workers kept by districts and regions.

### Maintenance and sustainability

This ongoing phase started in the middle of 2013. After rollout of the systems, many activities are ongoing at different levels to strengthen sustainability of the systems in the maintenance phase.

#### Strengthen sustainability for running HRHIS and TIIS

During the rollout phase, it was realized that some districts and training institutions were losing trained staff due to staff turnover. Therefore, MOHSW made an agreement with one of the universities in Tanzania to train all students who undergo the Health Systems Management degree programme (production of health secretaries) before the students go out for field training, as health secretaries are the main users of HRHIS and TIIS on the ground.

HRHIS/TIIS operational trainings have been conducted as separate modules in their programme, and 260 students were trained in 2013 and 2014.

#### Integration of HRHIS/TIIS operation and maintenance into different strategies and guidelines

This action is very important to ensure fund allocation for sustainability of HRHIS and TIIS. The issue of maintenance and proper use of the systems was integrated into the e-Health strategy [20] under MOHSW ICT unit. Moreover, the HRH Strategic Plan 2014–2019 is also capturing the issue of HRHIS/TIIS sustainability. Improvement of data coverage and utilization of data collected through HRHIS and TIIS is clearly stated in Strategic Objectives 1 in the strategic plan [21].

#### Integration of HRHIS/TIIS with the national HMIS database (DHIS2)

MOHSW in Tanzania is strengthening its HMIS, which includes the adoption of District Health Information System version 2 (DHIS2) software as the national HIS data warehouse. All routine health data from health facilities are reported to the national DHIS2 server at the district level. Since all systems are centralized at the MOHSW server, DHIS2 software is linked to HRHIS to import aggregate data for calculation of HRH-related indicators based on HMIS data. The interoperability mechanism between these two systems is designed from the beginning since they are developed and supported by the same technical team. Moreover, HRHIS and TIIS are also developed on the same platform and designed to communicate with each other from the beginning of the project. Therefore, in our case, it will not jeopardize the effectiveness of HMIS.

To avoid the complication of data sharing among the existing systems and others that might be developed in the future, TIIS and HRHIS have a special module for data exchange using API, which does not require knowledge of how the other systems are coded prior to data exchange.

#### Integration of HRHIS/TIIS supportive supervision with other supportive supervision

Due to insufficient budget allocation for centralized supportive supervision of the two systems, an alternative way of conducting HRHIS and TIIS supportive supervision needs to be established. One option is to integrate it with regular supportive supervision of health services at the district and regional level. Some regions showed ownership of the system and started to take action in conducting HRHIS supportive supervision regularly. Another way is to integrate it with supportive supervision of DHIS2 and HMIS, as HRHIS and TIIS complement DHIS2 and HMIS.

To make sure that there is a continuity of system usage and data utilization at the district level, there is a need for health management teams to allocate resources for data collection, analysis and report generation including supporting e-Health initiatives under their disposal like HRHIS. CHMTs should organize trainings to update skills of system users, conduct data quality analysis and utilization workshops, produce reports for local usage and sharing with decision makers like councillors and make sure that they collect records for newly recruited employees and update the existing. We are of the opinion that, if these are done, districts will feel more responsible with the system and make sure it is sustained and used.

## Discussion

### Achievement

Based on the process and outcomes from the national rollout of HRHIS and TIIS, it is recognized that the Tanzanian health sector established useful, functional HRH information systems that can contribute to improving evidence-based planning, development and management of HRH and social welfare [20–23].

After 6 years of efforts and commitments of MOHSW, JICA and system developers, HRHIS and TIIS have been rolled out all over the country. MOHSW departments, government agencies, national hospitals and all 25 regions and 171 district local health authorities are using HRHIS for planning, development and management of HRH and social welfare. TIIS has been rolled out to all 154 health training institutions and universities that are producing health professionals in the country (82 public, 54 faith-based-organizations and 18 in the private sector), and it is well utilized for employees’ and students’ management, management of other resources and academic records keeping [24].

Data coverage in the private and public sectors has been increasing year by year since 2009 to date. As data coverage for both systems increased, information generated from the two systems started to be utilized at different levels. At the central level, information generated from HRHIS and TIIS is used for producing many documents. MOHSW started producing an annual HRH Country Profile in 2012 [22, 25]. It has also been utilized to produce the comprehensive HRH Production Plan 2014–2024 [26] as well as the third HRH and Social Welfare Strategic Plan 2014–2019 [13]. At the district level, district officials started using reports generated from HRHIS to fulfil the HRH information in the annual Comprehensive Council Health Plan. Moreover, data from HRHIS was used for midterm review of the Health Sector Strategic Plan 2009–2015 as the most reliable data source for HRH and social welfare in the country [27].

HRHIS has made it possible for each CHMT to realize its actual HRH personnel needs, to allocate and reallocate personnel to health facilities based on demand and expertise and to manage day-to-day HRH activities based on valid information. Reporting of HRH information to the MOHSW and the President Office, Public Service Management (POPSM) is done annually and has been a very time-consuming, costly and tedious job to do every year. Yet, the information submitted has been of low quality and difficult to assess on its manual format. The use of HRHIS has simplified and improved the reporting process, and the data can be validated centrally.

Data quality poses big challenges in the implementation of any computerized information system, HRHIS and TIIS included. The situation can worsen when users lack the necessary competences in using computerized systems or have little motivation for change. Once users had collected a big number of records, data incorrectness and inconsistency aspects were observed. In addressing these challenges, the implementers approached it both from technical and users perspectives. Users were given refresher trainings to make sure they collect and enter correct and relevant data; most of the entries to the systems were provided to users as drop-down options and some restrictions were implemented like avoiding unintentional deletion of data by users. In the technical perspective, a separate data quality check module was introduced which checks the validity of entered records based on pre-defined criteria. For example, to avoid double entries, salary numbers which are unique for each employee were made primary key; to avoid using the same name with different salary numbers, name and date-of-birth comparisons were made and an alert given to users, and to ensure proper recoding on date of birth, date of employment and promotions, these fields were compared and any inconsistency was notified to users for necessary action.

Pemba and others [28] reported the lack of a reliable and accurate single source for accessing data on admission or graduation numbers for all health professional programmes in Tanzania. TIIS thus serves as one-stop shop for information necessary for monitoring enrolment, production and projection of the health workforce. Sirili et al. recommended coordination and collaboration among various stakeholders in the health sector in addressing HRH crisis [29]; thus, we believe HRHIS and TIIS provide required information for informed decision-making in this endeavour.

### Key factors for success

Riley and others [6] suggested several issues to strengthen HRH information systems. It was suggested that more attention be paid to strengthening and reporting the features on 1) HRH data collection and collation from multiple sources; 2) deployment of HRH data across multiple sectors; 3) interoperability with other health information systems; 4) data cleaning, validation, and management for regular updates; and 5) usage of HRH data for policy and strategies for the training, deployment and retention of HRH. Additionally, a WHO report in 2010 stated the necessity of agreed standards and protocols; harmonization and alignment of HRH classifications, definitions and indicators; and continuous capacity building of system users [2].

The authors believe that HRHIS and TIIS are well designed and cover all recommendations and suggestions made in both WHO’s meeting report and the study done by Riley and others [2, 3]. The following factors have been identified as positively influencing the successful national rollout of HRHIS and TIIS:

- Using local experts for developing the software applications leading to cost reduction, easy maintenance and good availability of technical support.
- Involvement of system users in the software development process to understand the realities on the ground.
- Strong involvement of MOHSW sections and departments.
- Focusing on routine work of the system users, at health facilities, regions, districts and training institutions regarding HRH planning, development and management, rather than central management of HRH information
- Reliable technical support for the system users to strengthen system usage and obtain reliable HRH and social welfare workers’ information.
- Provision of data utilization training on HRH planning, development and management for improvement of information usage.
- Conducting close follow-up on system usage, data quality and continuous improvement of system application software.

The design of HRHIS and TIIS was developed based on the findings from the detailed situation analysis. The situation analysis was very helpful in discovering issues and problems of HRH information management in the field and in identifying needs and expectations of the system users.

### Challenges

Building consensus on functional and sustainable information systems for HRH among stakeholders was not easy in the development phase. Many thought that the introduction of computerized information systems was the solution for HRH and social welfare planning, development and management and did not pay much attention to other key elements of the information system such as data collection, data entry, updating information and utilization of information.

Knowing the exact number of existing HRH and social welfare workers and their expertise in the country was a major challenge in the pilot and rollout phases. As a baseline survey, the number of paper files kept by the system users was used as the denominator for data coverage. Then, information of HRH and social welfare workers was entered into HRHIS and TIIS, and the entered data was used as the numerator for calculation of data coverage.

After data collection and entry, we found that some districts entered the number of HRH and social welfare workers bigger than the denominator; as a result, it calculated the system data coverage as more than 100 %. This indicates that information of HRH and social welfare workers had not been updated or managed properly, providing that the numerator figures entered into the systems were correct.

Other challenges were identified during supportive supervision of the two systems. Although the systems were rolled out all over the country, some districts and institutions have no reliable Internet connection for official use, instead relying on someone’s modem to get online and update HR information. This situation slows down system usage since users have to spend a lot of time waiting for network response. Computer illiteracy among system users was also identified. Due to computer illiteracy and pre-conceived negative experience of system users, data entry and information management tasks were given to the system users who had computer skills.

Another challenge was the cost of maintenance for the two systems at the central level. Central server maintenance and centralized supportive supervision is costly and difficult to sustain under chronic shortage of financial resources at the central level. Therefore, it is necessary to consider integrating supportive supervision of HRHIS and TIIS with general supportive supervision in regions and districts or with supportive supervision of the DHIS2 or HMIS, as HRHIS and TIIS are modules of DHIS2 and HMIS [21].

### Lessons learned

To establish functional HRH information systems, it is necessary to establish a sustainable mechanism for data collection, data entry, updating information and utilization of information for HRH planning, development and management. We have learned that the framework of HRH information systems should be developed and shared with all stakeholders to come to a consensus on the HRH information systems at an early stage.

The successes and failures of health informatics projects have been argued in previous studies [12, 13, 30, 31]. The design–reality gap model is discussed in those studies. Heeks’s study [12] concluded that the design–reality gap model provides a conceptual basis on which to understand and address health information systems’ successes and failures, and the model can be utilized as an evaluation tool to underpin the failure and success of health information systems. The authors also think that findings from detailed situation analysis made the system design more realistic and accepted by different levels of system users. A HRH information system involves a series of actions, from data collection, entering, analysing, reporting and utilization of data for planning to development and management of HRH. Therefore, proper understanding of the needs and expectations of the system users and consideration of their daily routine work are very important when developing HRH information systems, rather than designing only from the central ministry perspective.

Many developing countries introduced commercial or open-source ready-made HRH information systems to build their HRH information systems [32–34]. However, based on past experiences, Tanzanian MOHSW did not choose to use commercial or open-source ready-made information systems since they need to be localized. However, it costs and consumes a lot of time for localization. Even if a system application is built for free and open source, upgrading and maintenance of the system needs to be done by a system developer, who often is not based in the country. On the other hand, if the system is built from scratch, the country can find the following advantages: the ownership of the system can be strengthened throughout the process, there is flexibility to reflect on the needs of system users and to make quick modifications which can reduce routine workloads on HRH planning and management and there is high availability of technical backup as technical resources are in the country.

There is no reliable data to compare the cost between introducing commercial or open-source ready-made software and developing one’s own software for HRH information systems from scratch. However, from looking at the achievement of the national rollout of HRHIS and TIIS, and MOHSW’s strong ownership of the two systems, the authors believe that MOHSW made the right choice for developing and rolling out their own computerized HRH information systems.

## Conclusions

This case study presents the experiences of the national rollout of HRH information systems in Tanzania. Establishment and national rollout of HRH information systems was not an easy task. It took 6 years to rollout all over the country with the involvement of various stakeholders. Due to efforts and commitments from all stakeholders, the two systems are now well recognized as the most reliable data source for HRH and social welfare workers and health training institutions in Tanzania.

Using local system developers and developing software from scratch may take more time and effort than introducing ready-made software to establish HRH information systems in developing countries. However, this study suggests that using local system developers to establish a country’s own information system and structures seems to have more advantages than disadvantages in the case of Tanzania, especially in minimizing the design–reality gap and establishing a realistic information system that matches with local needs and supports routine work for HRH planning, development and management.

If there are reliable system developers within the country and development partners who are willing to support, the Tanzanian approach can be one choice to establish effective and sustainable HRH information systems in a developing country. We hope that the findings will be useful for other developing countries, especially in Africa, that are planning to establish and rollout effective HRH information systems.

We recommend that empirical studies be done on this initiative to establish its benefits to the overall health system in terms of system change, minimizing management and administrative costs and how the success of this initiative can be used to inform scaling of other e-Health solutions developed in the context of low-income countries like Tanzania.

## Abbreviations

CHMT: Council health management team
DHIS2: District Health Information System version 2
DHRD: Department of Human Resource Development
DUT: Data utilization training
HMIS: Health Management Information System
HRH: Human resource for health
HRHIS: Human Resource for Health Information System
ICT: Information and communication technology
JICA: Japan International Cooperation Agency
MOHSW: Ministry of Health and Social Welfare
PDCA: Plan-Do-Check-Act
POPSM: President Office, Public Service Management
RHMT: Regional health management team
TIIS: Training Institution Information System
